# Fungal Biofilms

**DOI:** 10.1371/journal.ppat.1002585

**Published:** 2012-04-05

**Authors:** Saranna Fanning, Aaron P. Mitchell

**Affiliations:** Department of Biological Sciences, Carnegie Mellon University, Pittsburgh, Pennsylvania, United States of America; Duke University Medical Center, United States of America

## Introduction

Biofilms are a principal form of microbial growth and are critical to development of clinical infection. They are responsible for a broad spectrum of microbial infections in the human host. Many medically important fungi produce biofilms, including *Candida* [1], *Aspergillus* [2], *Cryptococcus* [3], *Trichosporon* [4], *Coccidioides* [5], and *Pneumocystis* [6]. In this review we emphasize common features among fungal biofilms, and point toward genes and pathways that may have conserved roles.

Biofilm cell communities are more resistant to antifungal drugs than planktonic cells. Contributing factors include biofilm structural complexity, presence of extracellular matrix (ECM), metabolic heterogeneity intrinsic to biofilms, and biofilm-associated up-regulation of efflux pump genes. The actual fold increase in resistance varies with both the drug and species. *Candida albicans* and *Candida parapsilosis* biofilms are relatively resistant to fluconazole, amphotericin B, nystatin, voriconazole, and others. *Aspergillus fumigatus* biofilms are relatively resistant to itraconazole and, to some extent, to caspofungin. Cryptococcal biofilms are unaffected by fluconazole and voriconazole, and biofilms of *Trichosporon asahii* display elevated resistance to amphotericin B, caspofungin, voriconazole, and fluconazole. Azole and amphotericin B therapies are ineffective against *Pneumocystis carinii* biofilms. Biofilm-associated resistance mechanisms have been characterized in *C. albicans* and *A. fumigatus* and include drug binding by ECM and production of persister cells [2], [7] (see supplementary references for this section in Text S1). Persister cells represent only a fraction of the population, and probably reflect its metabolic heterogeneity. These mechanisms may pertain to other fungi as well.

## Fungal Pathogen Biofilm Architecture

Biofilms are complex surface-associated cell populations embedded in an ECM that possess distinct phenotypes compared to their planktonic cell counterparts. Nutrients, quorum-sensing molecules, and surface contact are contributory factors. *C. albicans* biofilms are comprised primarily of yeast-form and hyphal cells, both of which are required for biofilm formation [1]. Formation is a sequential process involving adherence to a substrate (either abiotic or mucosal surface), proliferation of yeast cells over the surface, and induction of hyphal formation [1]. ECM accumulates as the biofilm matures, and seems to contribute to cohesion [8]. *C. albicans* biofilms form on numerous abiotic [9] and biotic surfaces [10]–[12]. In denture stomatitis, a combination of biotic mucosal (the host) and abiotic surface (the denture) biofilm formation exists [13]. Other *Candida* spp. including *C. tropicalis, C. parapsilosis*, and *C. glabrata* form ECM-containing biofilms but do not produce true hyphae [14].

*Aspergillus* biofilms can form both on abiotic and biotic surfaces [15]. The initial colonizing cells that adhere to the substrate are conidia. Mycelia (the hyphal form) develop as the biofilm matures [15]. ECM that binds the biofilm together [15] has been observed in vitro [15] and in vivo [16]. Hyphal organisation is different in the two forms of *A. fumigatus* biofilm infection: aspergilloma infections present an intertwined ball of hyphae; aspergillosis infections present individual separated hyphae [16]. Hyphae of *C. albicans* and of *A. fumigatus* can form pores or channels through biotic surfaces [17], [18].

The emerging fungal pathogen *T. asahii* forms biofilms comprised of yeast and hyphal cells embedded in matrix [4], as do those of *Coccidioides immitis* [5]. *C. neoformans* forms biofilms consisting of yeast cells on many abiotic substrates [3], and shed capsular polysaccharide forms the ECM. Although *C. neoformans* forms hyphae in the course of mating, no hyphae have been observed in *C. neoformans* biofilms to date. Similarly, *Pneumocystis* species do not produce hyphal structures as part of their biofilms [6]. Thus, hyphal formation is not a uniform feature of fungal biofilms.

## Genetic Determinants of Fungal Biofilm Formation

Transcription factors play fundamental roles in both positive and negative regulation of biofilm formation through regulation of hyphal formation and cell surface proteins responsible for adherence [1]. Bcr1, a C_2_H_2_ zinc finger transcription factor, is a critical determinant of *C. albicans* biofilm formation in all environments studied to date [10], [12], [13], [19]. Bcr1 seems to be a conserved regulator of biofilm formation, because the Bcr1 ortholog of *C. parapsilosis* is required for biofilm formation as well [20]. Ace2, another C_2_H_2_ zinc finger transcription factor, also contributes to *C. albicans* biofilm formation, probably through its role in adherence as well as hypha formation [21]. The *C. albicans* transcription factor Efg1, a global regulator of cell surface protein genes and hyphal formation [1], is required for biofilm formation as well. The orthologs or best hits of Bcr1, Ace2, and Efg1, including *C. glabrata* CAGL0E06116g, CAGL0M04323g, and CAGL0L01771g, and *C. parapsilosis* CPAG00564, CPAG00148, and CPAG00178, are good candidates for biofilm regulators in those species. Transcription factors with analogous roles to Bcr1, Ace2, and Efg1 of *C. albicans* in *A. fumigatus* may be identified amongst the 124 uniquely expressed or upregulated transcription factors identified in biofilm culture by Gibbons et al., 2011 [22]. The *A. fumigatus* transcription factor *LAEA,* a regulator of cell type and secondary metabolism gene clusters in *A. fumigatus*, is highly upregulated in biofilms [22], and it remains to be seen whether it may influence biofilm phenotypes.

Cell wall proteins are of particular interest in biofilm formation. Besides its expected role in adherence, the cell wall may have a sensory role that promotes adherence-induced responses [23]. Numerous cell wall protein genes that may function in these capacities are upregulated early in *C. albicans* and *A. fumigatus* biofilm formation [22], [24], [25]. *C. albicans* cell surface proteins have been reviewed authoritatively (see Text S1). Among the upregulated *A. fumigatus* surface proteins are the hydrophobins RodA, RodB, RodD, and RodE. *RODB* is thought to play the most crucial role with its expression increased over 4,000-fold in biofilm versus planktonic growth conditions [22]. Ten other putative adhesins have been identified by Gibbons et al., 2011 [22]. It is possible these *Aspergillus* proteins have functions analogous to known adhesins in *C. albicans.*

## Gene Expression Portrait of Fungal Biofilms

Biofilm cells have phenotypes distinct from planktonic cells, and this difference is reflected in greatest detail at the gene expression level. Detailed gene expression profiling comparisons, conducted in both *C. albicans* and *A. fumigatus,* have revealed substantial changes in gene expression between biofilm and planktonic cells [22], [26]. Changes in transcription factor expression is characteristic of *C. albicans* biofilm formation in vitro and in vivo [24]–[26], suggesting biofilm formation to be a highly regulated process. Similarly, almost 50% of the predicted transcription factors of *A. fumigatus,* including many with roles in asexual and sexual development, are upregulated in biofilms compared to planktonic cells.

Although biofilms are thought to include dormant cells, biofilms of *C. albicans* and *A. fumigatus* have increased expression of genes involved in protein synthesis. These genes encode ribosomal proteins, protein turnover, and translation factors as well as ribosomal proteins, indicating increased protein translation and ribosome production in biofilms to be a feature of biofilms [22], [25], [26]. If indeed biofilm cells are nutrient limited, these particular gene expression features may optimize recycling of cellular constituents.

Upregulation of multi-drug resistance transporter genes is common to *A. fumigatus* (*MDR1, MDR2, MDR4*) and *C. albicans* (*MDR1, CDR1, CDR2)* biofilms in vitro [22]. *C. albicans MDR1* and *CDR2* are upregulated in in vivo biofilms, as is *PDR16*, which is increased in fluconazole-resistant cells that overexpress *CDR1* and *CDR2* [25]. Phase dependency of these transporters exists in vivo for *C. albicans CDR1* and in *A. fumigatus* for *MDR4* [25], [27]. Additionally, ergosterol gene expression may account for increased drug resistance of biofilms. Genes involved in sterol biosynthesis are upregulated in *A. fumigatus* and *C. albicans* biofilms [22], [25], [26]. Increases in *ERG* gene expression as well as multi-drug resistance transporters has been correlated with increased azole resistance in *C. albicans* patient isolate samples, though their contribution to biofilm-specific azole resistance has not been detected in mature biofilms (see Text S1).

Increased expression of adherence genes is also a property of biofilm cells. *ALS1* is the most upregulated of the known adherence genes of *C. albicans* under biofilm conditions. Garcia-Sanchez et al. (2004) [26] highlight that the *ALS* genes are differentially expressed in biofilms and have autonomous contributions in the biofilm transcriptome. Nett et al. (2009) [25] observed differential expression of *ALS* genes at different stages of biofilm formation and potential for overlap of function in vivo. A similar pattern of differential adhesin expression is seen in vitro in the *A. fumigatus* biofilm environment [22]. The inducing signal for biofilm adherence genes is clearly an area of interest as a basic biological question as well as a direction for prospective therapeutic development.

A significant number of primary metabolism genes, including those for amino acid synthesis, in particular sulfur amino acid biosynthesis, and nucleotide synthesis, are upregulated in *C. albicans* biofilms in vitro [24], [26] and in vivo [25], relative to in planktonic cells in vitro. Many are regulated by *GCN4*, a transcriptional activator required for biofilm formation [26]. Genes involved in amino acid metabolism are also upregulated in *A. fumigatus* biofilms including amino acid permeases, transporters, and amino peptidases. Secondary metabolism gene upregulation is significant in *A. fumigatus* biofilms, possibly due to upregulation of *LAEA*, a secondary metabolism regulator [22]. Altered metabolic gene expression may reflect nutrient limitation, but the rapid kinetics of induction (in *C. albicans* at least [24]) may reflect a different regulatory signal.

Many cell wall biogenesis genes are induced in the biofilm environment. Altered expression of genes for β-1,3 glucan synthesis and modification are features of in vivo *C. albicans* biofilms including *FKS1*, *BGL2*, and *XOG1* [25]. Given the connection between the β-glucan pathway and biofilm matrix production, these may also contribute to ECM production. Nett et al. (2009) [25] highlight downregulation of β-1,3 glucan degrading enzymes in 24-hour biofilms and suggest this functions in glucan conservation for matrix production. In contrast, altered expression of α- and β-1,3 glucan synthesis genes is not observed in *A. fumigatus* biofilms. Although it is not directly reflected by the expression of polysaccharide synthase genes, the presence of α-1,3 glucan, galactosaminogalactan, and galactomannan in the mycelial extacellular matrix is correlated to the aerial growth of the mycelium of *A. fumigatus* [16]. Expression of more than 50% of cell wall genes investigated in *A. fumigatus* is, however, altered in the biofilm habitat, including upregulation of the *ROD* genes. Thus, these two organisms both restructure their cell surfaces in biofilms, though they may use different mechanisms to achieve that outcome.

## Mating Type and Fungal Biofilms

Genetic exchange is a feature of bacterial biofilms, mediated in part by extracellular DNA. Although extracellular DNA has been detected in *C. albicans* biofilms [28], the main mechanism of biofilm-associated genetic exchange involves mating and cell fusion. Most biofilm studies have been conducted with nonmating a/α cells, but biofilm formation of the mating-capable cell types, a/a and α/α, has revealed a unique regulatory pathway intimately tied to pheromone signalling. In order to mate, *C. albicans* must go through a switch from the white to opaque cell type. Upon switching, α/α opaque cells release a mating pheromone that induces a mating response in a/a opaque cells and vice versa. Pheromone release also induces an adhesive phenotype among the mating-incompetent a/a white cells [29], leading to mixed biofilm formation and ultimately mating [30].

Notably, genes upregulated specifically in white cells in response to pheromone exposure specify primarily cell wall and surface proteins [29]. Several of these genes contribute to a/a-α/α biofilm formation [29]. The configuration of the mating type locus also seems to affect global biofilm properties [30], which may result from distinct signalling pathways [30]. If *C. albicans* has distinct ways to make a biofilm, it seems likely that other fungi will as well.

## Perspective

Fungal biofilms reflect a range of architectures. Regulators of biofilm formation may be conserved even among disparate biofilm architectures. From detailed analysis in *C. albicans* and *A. fumigatus*, there are numerous candidate genes that could be investigated in other biofilm-forming fungi. In addition to hyphal gene expression, characteristic biofilm gene expression patterns include increased expression of transcription factors and protein synthesis genes. Differential adhesin expression, upregulation of cell wall genes, and increased primary metabolism are features of the biofilm environment. Studies of mating pheromone effects on adherence highlight how a small portion of biofilm constituents can have a significant impact on biofilm formation. The presence of highly drug tolerant persister cells in biofilms (discussed above) is another illustration of the contribution of cell heterogeneity to overall biofilm properties. How other heterogeneous properties among biofilm cells may contribute to the overall development and integrity of pathogenic fungal biofilms will be an interesting question for future research.

**Figure 1:**
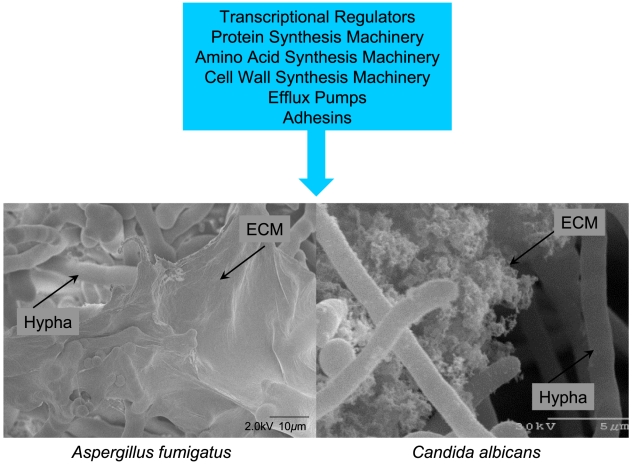
Common features of fungal biofilms. Gene expression has been compared between planktonic cells and biofilm cells of both *A. fumigatus* and *C. albicans*. The major functional categories of genes upregulated in biofilms are summarized in the blue box. The micrographs below show a cryo SEM view of an *A. fumigatus* biofilm (left; Stephanie Guadagnini, Anne Beauvais, and J.P. Latge, Institut Pasteur, Paris, France) and a scanning EM view of *C. albicans* in vitro biofilm cells (right; Fanning, Suhan, and Mitchell, unpublished). In both cases, extracellular matrix (ECM) is evident.

## Supporting Information

Text S1 Supplementary references
(PDF)
