# Supplementary material for: Fungal Biofilms
Source: PLoS Pathog. 2012 Apr 5;8(4):e1002585. doi: 10.1371/journal.ppat.1002585 (PMC3320593; doi:10.1371/journal.ppat.1002585)
Supplement: Text S1 — Supplementary references (PDF) [file ppat.1002585.s001.pdf]

Supplementary References

- They are responsible for a broad spectrum of microbial infections in the human host [1]
- *Candida albicans* and *Candida parapsilosis* biofilms are relatively resistant to fluconazole, amphotericin B, nystatin, voriconazole and others [2].
- *Aspergillus fumigatus* biofilms are relatively resistant to itraconazole and, to some extent, to caspofungin [3].
- Cryptococcal biofilms are unaffected by fluconazole and voriconazole [4] and biofilms of *Trichosporon asahii* display elevated resistance to amphotericin B, caspofungin, voriconazole, and fluconazole [5].
- Azole and amphotericin B therapies are ineffective against *Pneumocystis carinii* biofilms [6].
- Biofilm-associated resistance mechanisms have been characterized in *C. albicans* and *A. fumigatus* and include drug binding by ECM [7] and production of persister cells [8, 9].
- *C. albicans* transcription factor Efg1, a global regulator of cell surface protein genes and hyphal formation [10] is required for biofilm formation as well [11, 12].
- *C. albicans* cell surface proteins have been reviewed authoritatively [13]
- Increases in *ERG* gene expression as well as multi-drug resistance transporters has been correlated with increased azole resistance in *Candida albicans* patient isolate samples [14], though their contribution to biofilm-specific azole resistance has not been detected in mature biofilms [15].

1. Hall-Stoodley, L., J.W. Costerton, and P. Stoodley, *Bacterial biofilms: from the natural environment to infectious diseases.* Nat Rev Microbiol, 2004. **2**(2): p. 95-108.
2. Kuhn, D.M., et al., *Antifungal susceptibility of Candida biofilms: unique efficacy of amphotericin B lipid formulations and echinocandins.* Antimicrob Agents Chemother, 2002. **46**(6): p. 1773-80.
3. Mowat, E., et al., *The characteristics of Aspergillus fumigatus mycetoma development: is this a biofilm?* Med Mycol, 2009. **47 Suppl 1**: p. S120-6.
4. Martinez, L.R., E. Christaki, and A. Casadevall, *Specific antibody to Cryptococcus neoformans glucurunoxylomannan antagonizes antifungal drug action against cryptococcal biofilms in vitro.* J Infect Dis, 2006. **194**(2): p. 261-6.
5. Di Bonaventura, G., et al., *Biofilm formation by the emerging fungal pathogen Trichosporon asahii: development, architecture, and antifungal resistance.* Antimicrob Agents Chemother, 2006. **50**(10): p. 3269-76.
6. Cushion, M.T. and M.S. Collins, *Susceptibility of Pneumocystis to echinocandins in suspension and biofilm cultures.* Antimicrob Agents Chemother, 2011. **55**(10): p. 4513-8.

7. Nett, J.E., et al., *Genetic basis of Candida biofilm resistance due to drug-sequestering matrix glucan.* J Infect Dis, 2010. **202**(1): p. 171-5.
8. Beauvais, A. and F.M. Muller, *Biofilm Formation in Aspergillus fumigatus*, in *Aspergillus fumigatus and Aspergillosis*, J.P. Latge and W.J. Steinbach, Editors. 2009, ASM Press: Washington DC. p. 149-157.
9. LaFleur, M.D., C.A. Kumamoto, and K. Lewis, *Candida albicans biofilms produce antifungal-tolerant persister cells.* Antimicrob Agents Chemother, 2006. **50**(11): p. 3839-46.
10. Finkel, J.S. and A.P. Mitchell, *Genetic control of Candida albicans biofilm development.* Nat Rev Microbiol, 2011. **9**(2): p. 109-18.
11. Ramage, G., et al., *The filamentation pathway controlled by the Efg1 regulator protein is required for normal biofilm formation and development in Candida albicans.* FEMS Microbiol Lett, 2002. **214**(1): p. 95-100.
12. Sohn, K., et al., *EFG1 is a major regulator of cell wall dynamics in Candida albicans as revealed by DNA microarrays.* Mol Microbiol, 2003. **47**(1): p. 89-102.
13. Chaffin, W.L., *Candida albicans cell wall proteins.* Microbiol Mol Biol Rev, 2008. **72**(3): p. 495-544.
14. White, T.C., *Increased mRNA levels of ERG16, CDR, and MDR1 correlate with increases in azole resistance in Candida albicans isolates from a patient infected with human immunodeficiency virus.* Antimicrob Agents Chemother, 1997. **41**(7): p. 1482-7.
15. Mukherjee, P.K., et al., *Mechanism of fluconazole resistance in Candida albicans biofilms: phase-specific role of efflux pumps and membrane sterols.* Infect Immun, 2003. **71**(8): p. 4333-40.
